# Case Report: Formation of 3D Osteoblast Spheroid Under Magnetic Levitation for Bone Tissue Engineering

**DOI:** 10.3389/fmolb.2021.672518

**Published:** 2021-06-21

**Authors:** Iñigo Gaitán-Salvatella, Edgar Oliver López-Villegas, Patricia González-Alva, Fernando Susate-Olmos, Marco Antonio Álvarez-Pérez

**Affiliations:** ^1^ Tissue Bioengineering Laboratory, Postgraduate Studies and Research Division, Faculty of Dentistry, National Autonomous University of Mexico (UNAM), México City, Mexico; ^2^ Escuela Nacional de Ciencias Biológicas, IPN, México City, Mexico; ^3^ Instituto de Fisiología Celular, México City, Mexico

**Keywords:** cell culture techniques, biocompatibility, ALP activity, bone tissue engineering, cellular spheroids

## Abstract

Skeletal reconstruction is necessary in cases of bone defects created by tumors, trauma, and abnormalities. Regeneration of bone defects remains a critical problem, and current approaches are based on biocompatible scaffolds. Spheroids represent a simple 3D system since no supporting material is required for cell growth. Different techniques are used to generate spheroids, such as hanging drop, low-attachment plates, and magnetic nanoparticles. The idea of using magnetic nanoparticles is to cross-link through cell membrane overnight to create complex 3D cellular spheroid by using magnets to guide the cellular response. Herein, the current study aimed to achieve 3D human fetal osteoblast (hFOB) spheroid under magnetic levitation. Formation of 3D spheroid culture under magnetic levitation was evaluated by cell viability at 3, 7, and 14 days. Morphology of the 3D hFOB spheroid was analyzed by SEM and fluorescence microscopy and the differentiation towards mineralized lineage by ALP assay, qPCR, and alizarin red staining. The cell viability indicated that the 3D hFOB spheroid still viable after 14 days of culture. ALP assay, qPCR analysis expression of Col1, ALP, and Itg-β1 molecules, and calcium deposition with alizarin red showed a high level of bioactivity of the 3D hFOB spheroid. SEM images allowed the morphological analysis of the 3D microtissue-like spheroid with the presence of matrix deposition. These results indicate that magnetic levitation culture enables 3D stable osteoblast spheroids and could be a promising strategy for engineering application in the 3D construct in surgery regeneration of mineralized tissue.

## Introduction

Bone defects resulting from trauma, disease, surgery, or congenital malformations are a significant health problem worldwide. The current gold standard for bone defect repair is still using auto- and allograft in an orthopedic trauma surgery (De Long et al., 2007). This strategy has drawbacks as secondary surgery, donor-site morbidity, insufficient availability, disease-transfer risk, and potentially negative immune response (Janssen et al., 2014; Fernandez de Grado et al., 2018). The problems associated with transplanted grafts have raised interest in bone tissue engineering in searching and improving the design of three-dimensional (3D) cellular constructs. This growing interest in culturing adherent cells for developing a 3D construct is one of the most important issues for tissue engineering. It opens a new research area in 3D cell culture techniques because there is a significant difference between the cellular phenotype and biological response of cells cultured in two-dimensional (2D) vs. 3D cell culture systems (Kelm et al., 2003; Park et al., 2018).

Traditionally, 2D cell culture systems are a standard method for proliferating cells in which the cells grow as a monolayer and do not fully reflect tissues’ physiology due to some limitations in mimic *in vivo* multicellular conditions. In contrast, the 3D culture could mimic the microenvironment’s specificity during embryogenesis, morphogenesis, and organogenesis (Pampaloni et al., 2007; Laschke and Menger, 2017). Thus, research has been directed towards 3D systems construct as promising strategies in tissue regeneration, particularly in the study of the formation of multicellular aggregates or spheroids, due to their self-assembly nature and conserve viability. Also, the system supports new and healthy functional 3D tissue formation and has gained attention (Mironov et al., 2009). Spheroids cultures represent a simple 3D system, where no scaffold or supporting material is required for 3D cell growth and has significant advantages as facilitate cell-cell and cell-matrix interaction networks, provide a similar physicochemical environment, secrete cytokines, chemokines, and angiogenic factors and maintain intrinsic phenotypic properties identical to the *in vivo*, and tissue morphology and function is different from that of cells in the monolayer culture system (Griffith and Swartz, 2006; Fennema et al., 2013)

Various methods are available for generating 3D spheroids. They include culture in spinner flasks, the hanging drop method, low-attachment plates, and microfabrication (Lim and Chang, 2008; Yu et al., 2010; Haycock, 2011; Anada et al., 2012; Todorov and VadeBoncouer, 2014). However, all these methods report that the cell aggregates’ size (spheroids) impacts cell responses for the *in vivo* microenvironment stimulation. Hence, to broaden their tissue engineering applications, they still need further improvement and validation (Kunz-Schughart et al., 1998; Asthana and Kisaalita, 2012).

Recently, hybrid approached so-called guided self-assembly have been developed (Tasoglu et al., 2014). One of the guided self-assembly-based technology to mimic the biological effects of weightlessness is the magnetic levitation technique. This 3D culture system is based on culture cells mixed with magnetic nanoparticles (MNP) and subjected to magnetic force (Bumpers et al., 2015). This condition induces the geometry change of cell mass and promotes contact between cells, leading to cell aggregation in an air/liquid interface during spheroid formation through negative magnetophoresis (Edmondson et al., 2014). Magnetic levitation 3D system was reported using hydrogels containing gold and Fe_3_O_4_ nanoparticles that are self-assembled that bind to the cell surface to magnetize and aggregate them to produce 3D spheroid (Souza et al., 2010). Other similar methodologies include using Fe_3_O_4_ magnetic nanoparticles encapsulated onto PLGA microparticles for 3D spheroid tumor formation (Lee et al., 2011). The iron oxide-containing hydrogel is commercially available as NanoShuttles (NS); they consist of gold, iron oxide, poly-l-lysine, and work by electrostatically attaching to the cell membranes. The charged cells can then levitate over any stiff substrate by using neodymium magnetic excitators; the cells aggregate towards the air-liquid interface and work with each other to build a larger 3D structure that involves protein synthesis such as collagen, fibronectin, and laminin (Tseng et al., 2013). This magnetic levitation system has been reported as non-toxic and does not affect proliferation or induce any inflammatory response by cultured cells and is a well-established system culture onto tumor cell lines and primary cells as endothelial, smooth muscle, hepatocytes, and fibroblast (Souza et al., 2010; Tseng et al., 2014; Tseng et al., 2018; Natânia de Souza-Araújo et al., 2020; Urbanczyk et al., 2020). Thus, the reported technique is an alternative to both scaffold-based and scaffold-free 3D cell culture systems. For this reason, this work aimed to develop a scaffold-free facile production of 3D human fetal osteoblasts spheroids by magnetic levitation system and evaluated the biological response for bone tissue engineering applications.

## Materials and Methods

### Cell Culture

Human fetal osteoblast cells (hFOB, 1.19 ATCC CRL-11372) were cultured in 75 cm^2^cell culture flasks containing a 1:1 mixture of Ham’s F12 Medium Dulbecco’s Modified Eagle Media (DMEM, Sigma-Aldrich, St. Louis, MO, United States), supplemented with 10% fetal bovine serum (FBS, Biosciences, CA, United States ), 2.5 mM L-glutamine and antibiotic solution (streptomycin 100 μg/ml and penicillin 100 U/ml, Sigma-Aldrich, MO, United States). The cell cultures were incubated in a 100% humidified environment at 37°C in an atmosphere of 95% air and 5% CO_2_. hFOB on passage 2–6 were used for all the experimental procedures. For osteogenic differentiation, the hFOB cultures were grown in an osteogenic medium composed of 50 µM of ascorbic acid, 10 mM of glycerol phosphate, and 10^−7^ M of dexamethasone.

### Spheroids Development by Magnetic Levitation Culture

For hFOB spheroid formation, a 3D Bio Assembler TM System (Nano3D Biosciences, Inc.,) was used. Briefly, hFOB cell monolayer with 70–80% confluence was rinsed with saline phosphate buffer solution (PBS) and incubated overnight with 100 μL of the NanoShuttle solution of magnetic nanoparticles (NanoShuttleTM-PL). The NanoShuttle is a nanoparticle assembly of iron oxide (Fe_2_O_3_) and gold (Au) nanoparticles cross-linked with poly-l-lysine to promote magnetic association with the osteoblast cell membrane during a static overnight incubation. The next day, hFOB magnetized cultures were washed with PBS to remove off NanoShuttle nanoparticles excess, and trypsinized and seeded into an ultra-low attachment 96-well plate at a concentration of 5 × 10^4^ cells/well. Immediately and afterward, the 96-well plate was placed atop a magnetic drive of 96 neodymium magnets (0.0625″ OD, Nano3D Biosciences), the hFOB cells magnetically levitated, and the magnetic force guided them, and they aggregated within hours. The hFOB cells stay in levitation just below the meniscus at the center of the well, where they self-assemble into spheroids. The hFOB levitated spheroids were incubated in a 100% humidified environment at 37°C in an atmosphere of 95% air and 5% CO_2_ until analysis at indicated time points.

### Human Fetal Osteoblast Spheroid Cell Proliferation

The cell viability reagent CCK-8 determined the metabolic activity of hFOB spheroid; based on the ability of the dehydrogenated enzyme to reduce a tetrazolium salt 2-(2-methoxy-4-nitrophenyl)-3-(4- nitrophenyl)-5-(2, 4-disulfophenyl)-2H-tetrazolium, monosodium salt to a soluble orange product in the cell culture medium. First, we compare the cell viability of hFOB seeded in 2D tissue culture plates against 3D hFOB magnetic induced spheroid after 1, 7, and 14 days of culture. Then we compare the viability of the 3D hFOB spheroid after 3, 7, and 14 days of culture under magnetic levitation with and without the presence of osteogenic media. After each established time, 10 μl of the CCK-8 solution was added to the cell cultures and incubated at 37°C for 6 h. Then, 100 μl of the culture medium was removed and placed in a 96-well plate to obtain the optical density at a wavelength of 450 nm with an ELISA plate reader (ChroMate, AWARNESS). The experiments were conducted in triplicate.

### Human Fetal Osteoblast Spheroid Viability and Morphology Analysis

The viability of hFOB spheroid was analyzed by fluorescence microscopy. hFOB spheroid was incubated with CellTracker™ Green CMFDA (5-chloromethylfluorescein diacetate) in phenol red-free medium at 37°C for 30 min. Subsequently, the spheroid was washed carefully three times with PBS and incubated for 4 h in a complete medium. hFOB spheroids were observed using LED base epifluorescence microscopy (model T670Q-PL-FLLED-B, AMSCOPE).

The hFOB spheroid morphology was evaluated by optical and by scanning electron microscopy (FE-SEM 7600F, JEOL) using a 15 kV acceleration. For SEM analysis, after 14 days with and without the presence of osteogenic media incubation, the hFOB spheroid was washed three times with PBS, fixed with 2% glutaraldehyde overnight, washed twice with PBS, and then dehydrated with a graded series of ethanol (25–100%); finally, samples were subjected to critical point drying. The samples were sputter-coated with a thin layer of gold-palladium and examined by SEM. For optical images, hFOB spheroid was fixed with 4% paraformaldehyde (PFA), washed twice with PBS, and once with deionized water. Finally, spheroids were eventually observed under light microscopy (model T670Q-PL-FLLED-B, AMSCOPE).

### Alkaline Phosphatase Activity Assay

Alkaline phosphatase (ALP) enzymatic assay was measured using a colorimetric method based on the conversion of p-nitrophenyl phosphate into p-nitrophenol in the presence of ALP (ABCAM). hFOB spheroid after 3, 7, and 14 days of culture were washed gently with PBS, followed by incubated with 100 μl of ALP assay buffer for lysate the spheroids. Then, the lysates (50 µl) were added in a 96-well-plate adjusted to 80 µl with assay buffer, and then 50 µl of 5 mM of p-nitrophenol (p-NP) solution were added. The well plate was incubated protected from light at 25°C for 60 min, and the reaction was stopped by the addition of 20 μl stop solution. The colorimetric reaction was presented as absorbance and was read at 405 nm with an ELISA plate reader (ChroMate, AWARENESS Technology).

### Alizarin Red Staining

Osteogenesis mineralization of 3D hFOB spheroid was determined by alizarin red staining (ARS) using an osteogenesis quantization assay kit (Millipore) after 14 days of culture. The 3D hFOB spheroid’ positive red cells were observed by inverted optical microscope after alizarin red staining to analyze the extracellular matrix. For quantitative analysis of ARS staining, hFOB spheroids were transferred to a 1.5 ml microcentrifuge tube, incubated with 10% acetic acid for 30 min, and then heated at 85°C for 10 min. Samples were then cooled for 5 min on ice and centrifuged at 13,000 rpm for 10 min, and neutralized with 10% ammonium hydroxide. The concentration of ARS was determined by correlating the absorbance of the experimental samples with a standard curve of known ARS dye concentrations.

### Real-Time Quantitative PCR

Total RNA was extracted from 3D spheroids 14 days of culture under magnetic levitation with and without the presence of osteogenic media by using TRIzol reagent (Invitrogen, Carlsbad, CA, United States ). The RNA concentration was measured by using The Qubit® RNA BR (Broad-Range) Assay Kit (Thermo Fisher Scientific) in a Qubit® Fluorometer 2.0 (Invitrogen). For cDNA synthesis, 1 µg of total RNA was reverse transcribed using the ImProm-II Reverse Transcription System (PROMEGA). The reaction was performed following the protocol of amplification recommended by the manufacturer, in brief: anneal at 25°C for 5 min, extension temperature 42°C for 60 min, and a final step of heat inactivation at 70°C for 15 min. For qPCR analysis, the cDNA was diluted 1:10. The qPCR reaction was performed in 20 µL volume using the Forget-Me-Not™ EvaGreen® qPCR Master Mix (Biotium), and the program conditions were as follow: 95°C, for 2 min, 45 cycles of 95°C, 10 s and 60°C, 30 s, finally the dissociation curve was obtained by increasing the temperature from 60 to 97°C. The primers were designed using the Primer3Plus program and synthesized by SIGMA. Primer sequences for alkaline phosphatase (ALP): Fw CGA​CCA​GAC​GTG​AAT​GAG​AG, Rv GCT​ACG​AAG​CTC​TGC​TCC​TG; for collagen 1 (Col 1): Fw GAG​AGC​ATG​ACC​GAT​GGA​TT, Rv ATG​TAG​GCC​ACG​CTG​TTC​TT; for integrin β1 (Itgβ1): Fw GGT​CCA​ACC​TGA​TCC​TGT​GT, Rv GAA​CAA​TTC​CAG​CAA​CCA​CA; and for glyceraldehyde 3-phosphate dehydrogenase (GAPDH): Fw GCA​TCC​TGG​GCT​ACA​CTG​AG, Rv TGC​TGT​AGC​CAA​ATT​CGT​TG. The qPCR reaction was performed using MyGo Pro Real Time PCR system (IT IS International, Ltd., Middlesbrough, United Kingdom). Each sample was tested in triplicate. Expression results of target genes were normalized against GAPDH.

### Statistical Analysis

In order to compare the statistical differences between experimental and control groups, different tests were carried out to demonstrate some existing or non-existent relationships. First, a normality test was performed on both Kolmogorov-Smirnov and Shapiro-Wilk, for the case of both plates (3D culture and 2D monolayer culture) and among the media, the test resulted in a non-normal distribution, followed by a nonparametric test of Mann-Whitney U. To evaluated ALP activity, the one-way ANOVA statistical analysis was performed, with Tukey’s *post-hoc* test. For alizarin red analyses of the Student’s t-test were performed. All tests were carried out in the statistical program IBM SPSS statistics version 23 and with a value of *p*< 0.05 considered to determine statistically significant differences.

## Results and Discussion

**FIGURE 1:**
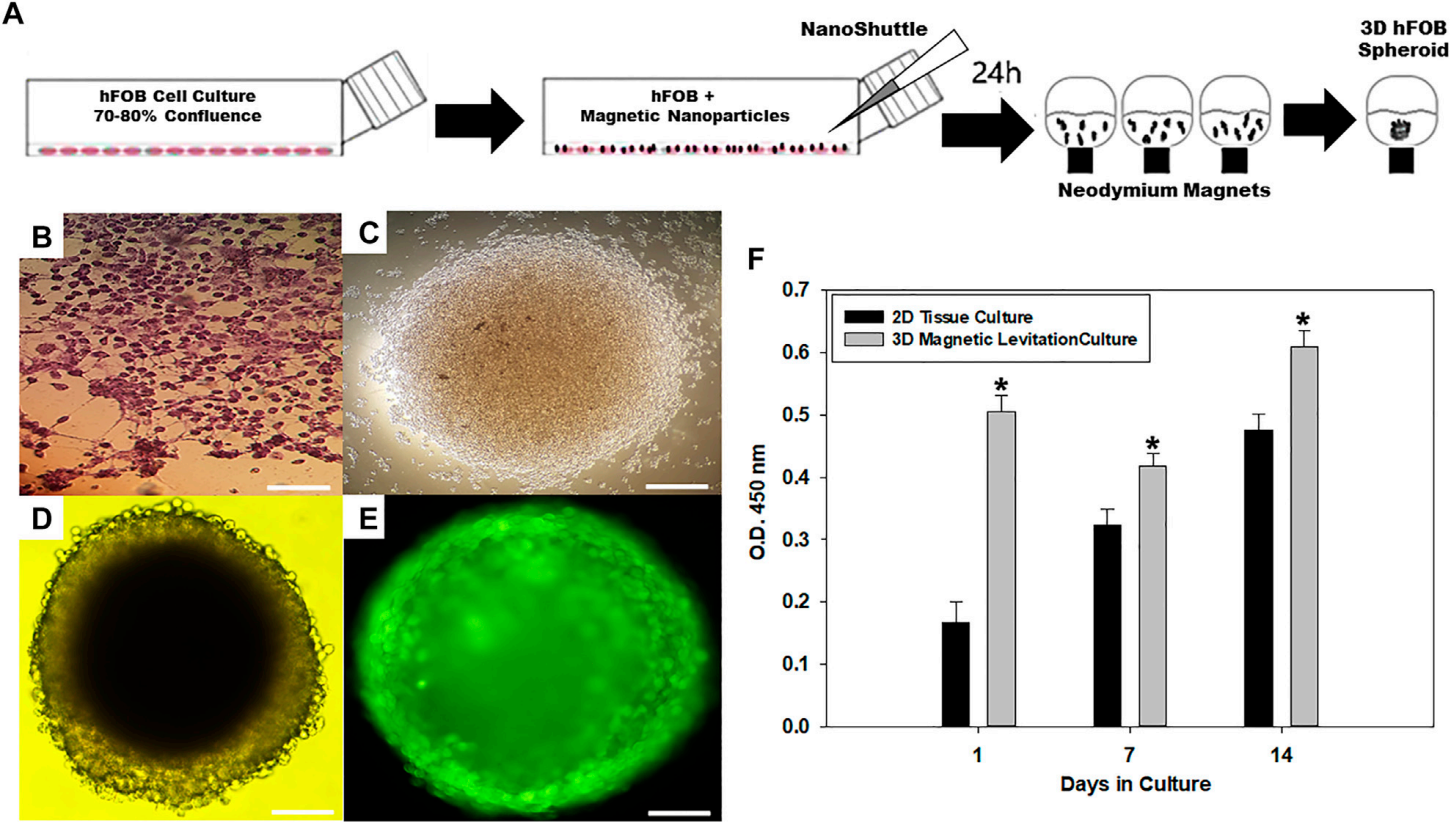
**(A)** Schematic of magnetic levitation technique. A confluent flask of hFOB cells (75–80%) was incubated with NanoShuttle overnight to allow for cell membrane-binding of the magnetic nanoparticles. The next day, the cells were seeded onto 96-well plate placed atop a magnetic drive of 96 neodymium magnets (0.0625″ OD, Nano3D Biosciences), the magnetic field influencing the hFOB cells to form an air–liquid interface and guide them to aggregate within hours of levitation to form the 3D Spheroid. **(B)** Optical micrograph of 2D tissue culture plate. **(C)** 3D hFOB spheroid after 3 h of magnetic levitation culture. **(D)** 3D hFOB spheroid after 24 h of magnetic levitation culture. **(E)** Fluorescence micrograph of 3D hFOB spheroid was performed to confirm the spheroid viability after 24 h of magnetic levitation culture. **(F)** Cell viability activity of 2D hFOB culture in comparison with 3D hFOB incubation with NanoShuttle and exposure to neodymium magnets after14 days. Group means were determined, and the analysis done using Mann–Whitney U test, asterisks (*) indicate significant differences at *p-*value< 0.05; error bars show standard deviation. Scale bar = 20 µm.

Bone grafts are utilized in a wide array of clinical settings to augment bone repair and regeneration. Currently, bone tissue engineering (BTE) has focused on monolayer cellular models that brought significant contributions to the understanding the biological response of the screening of novel biocompatible materials and the analysis of cellular physiology onto bone defect repair (Torricelli et al., 2004). BTE recently searched for alternative treatment options for regenerate bone tissue as a cellular-based three-dimensional culture models (3D) approach. This approach could better mimic the microenvironment in 3D tissues, including cell communication and adhesion between cell-cell and cell-extracellular matrix (Elliott and Yuan, 2011). Herein, this study presents a set of experiments to propose a consolidated and straightforward methodology based on magnetic levitation to develop a three-dimensional (3D) spheroid from human fetal osteoblasts (hFOB) culture. The 3D spheroid was obtained by incubating the hFOB culture with magnetic nanoparticles (MNP) in agreement with previous studies (Souza et al., 2010). The interaction between MNP with hFOB was let for 24 h to achieve the formation of spheroids. This interaction resulted in the formation of microcellular aggregates under the action of the magnetic field (Figure 1).

A qualitative morphological study by optical microscopy of the 3D hFOB spheroid was carried out to investigate the cell aggregates. hFOB cells started to aggregate to form small clusters after 3 h of magnetic action (Figure 1C), and the majority of hFOB cells were arranged onto a compact round-shaped spheroid after 24 h (Figure 1D). The viability of the 3D hFOB spheroid was analyzed by fluorescence after 24 h of magnetic interaction. From the image, it can be seen that the cells are viable because the fluorophore stained the membrane of viable cells and allowed us to visualize in detail the cell-cell interaction and the multicellular aggregation (Figure 1E). This multicellular spheroid had an average size of ∼ 100 µm after 3 days of culture and reached ∼ 350 ± 25.2 µm after 14 days of culture. The results are in concordance with studies that report that 3D spheroids with a medium-size guarantee the diffusion of oxygen and nutrients in the central region of aggregates, maintaining the integrity of the spheroid, on the contrary, when the 3D spheroids have a large size around of more than 450 µm cause low diffusive transport of oxygen and nutrients into the densely agglomerated cells in the central region of aggregates causing a greater risk of developing hypoxic centers that leads to cell necrosis and disintegration of the spheroid (Lewis et al., 2016; Koudan et al., 2017; Nath et al., 2017). Moreover, the continuous increase in cell proliferation of 3D hFOB spheroid was evaluated by cell viability CCK-8 assay and compared against the 2D hFOB monolayer culture proliferation. The results showed that 3D hFOB spheroids have an increased cell proliferation after 14 days of culture, and the differences between 3D and 2D monolayer cultured cells were statistically significant (Figure 1F). The application of magnetic levitation to hFOB cultures is suitable for supporting cell-cell interactions and, most importantly, allows the proliferation. Similar to preliminary studies, the present study results demonstrated that MNP and magnetic levitation are not toxic to the cells and could be a strategy for producing many spheroids rapidly with a narrow size distribution (Qian et al., 2012; Lewis et al., 2017).

In BTE, the cell type and source are essential for gaining further insight into bone formation and the bone regeneration process. Primary cell cultures are the most widely used, usually obtained from normal human and rodent bone tissue, and osteosarcoma cell lines have been extensively reported (Harris et al., 1995). In the present study, we utilized primary adhesive cultures of the hFOB cell line. These cells are naturally involved in physiological regeneration processes and can undergo osteogenic differentiation *in vitro*. Moreover, hFOB is an immortalized, clonal human fetal cell line that is well-characterized as osteoprogenitor with minimal karyotype damage, even after multiple passages, making the cell line a promising candidate for use in tissue-engineering approaches to bone regeneration (Subramaniam et al., 2002; Yen et al., 2007).

**FIGURE 2:**
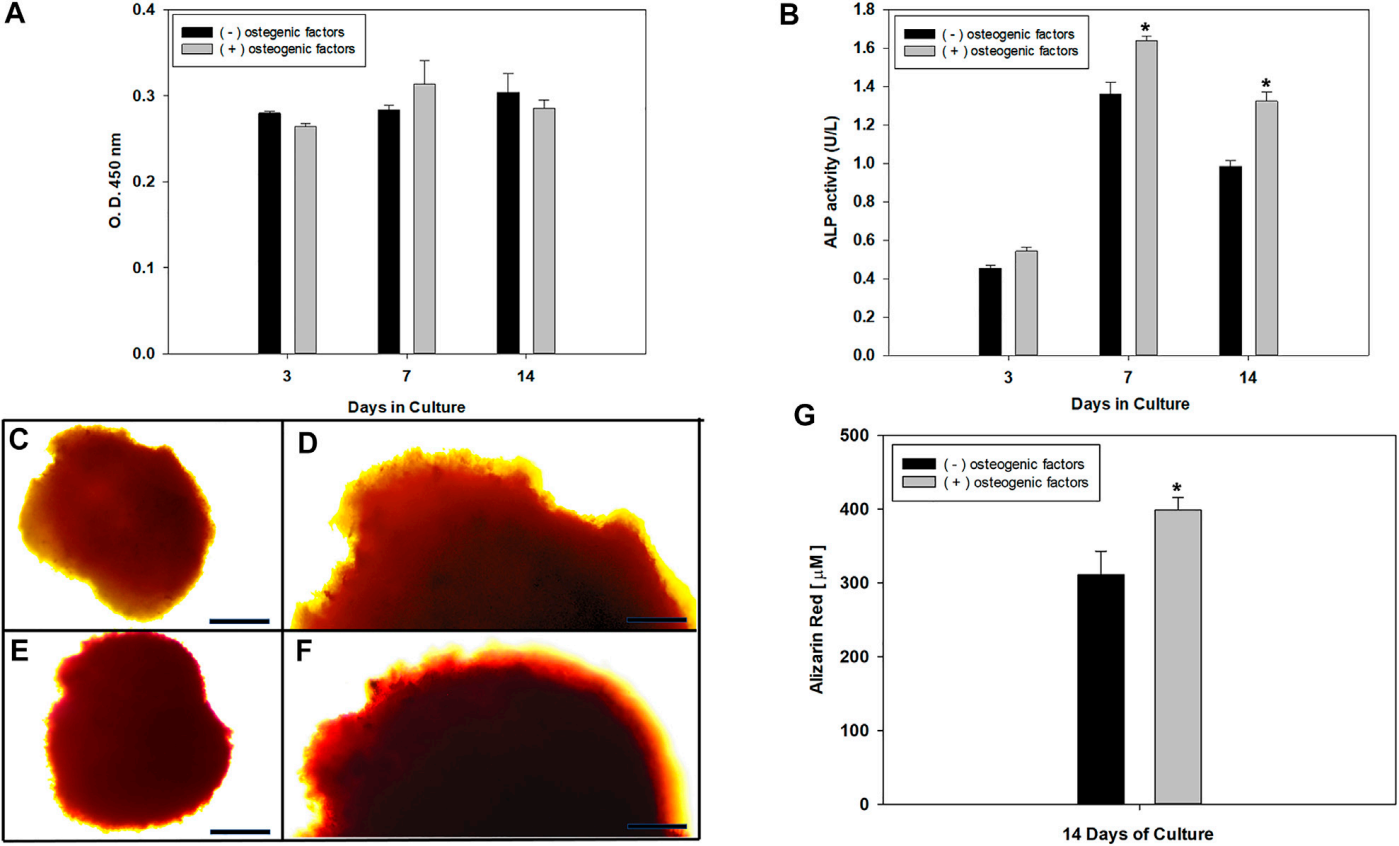
**(A)** hFOB spheroid viability after 3, 7, and 14 days of magnetic levitation culture treated with the presence and absence of osteogenic factors assessed using CCK-8 assay, analysis by using Mann–Whitney U test with *p-*value< 0.05; error bars show standard deviation showed no significant statistical differences between treatment. **(B)** ALP activity of 3D hFOB spheroid after 3, 7, and 14 days of culture in response to osteogenic signal and in comparison to 3D hFOB spheroid without any osteogenic signal. Analysis using ANOVA, One way with a *p*-value< 0.05 showed significant differences indicated by the asterisks (*). Alizarin Red S staining was assayed to visualize calcium deposition onto the 3D hFOB spheroids under light microscope. **(C and D)** ARS images shows the mild red-colored positive staining on the osteoblasts cells on the periphery of 3D control spheroid. **(E and F)** ARS images showed the dark-red colored onto the matrix spheroid of 3D spheroid under osteogenic factor. Scale bar = 100 µm. **(G)** Quantification reaction between calcium ions and ARS that indicate deposition of bone mineral (calcium deposits) by osteoblasts. Asterisks (^*^) indicate significant differences between conditions at p < 0.05 by Student's t-test.

Thus, we explore the comparison of 3D hFOB spheroid formation cultured with and without osteogenic factors and under magnetic levitation. It was intended as a promising strategy for regeneration and replacement of use in tissue-engineering biofabrication approaches to bone regeneration (Figure 2). The cell growth characteristics of the 3D hFOB spheroid showed an exponential growth after 14 days of culture. Also, the spheroid viability does not suffer any decrease indicating good proliferation without any cytotoxic response with no significant statistical differences between the presence or absence of osteogenic factors onto the magnetic levitation culture (Figure 2A). Moreover, the osteogenic differentiation of 3D hFOB spheroid was analyzed after 14 days of culture using specific markers such as ALP, COL-1, along with alizarin staining and calcium quantification. Alkaline phosphatase (ALP) enzyme is synthesized and secreted by active osteoblasts and considered a marker to reflect different aspects of osteoblast function and bone formation (Shetty et al., 2016). In our study, the ALP activity of 3D hFOB spheroid was measured by the enzymatic activity in the cell layer based on the hydrolysis of p-nitrophenyl phosphate to p-nitrophenol. Figure 2B shows the ALP activity at day 3; the activity was peaked and demonstrated a significantly increased time-dependent manner at 7 and 14 days after treatment with and without osteogenic factors. However, the ALP activity in the 3D spheroids cultured under the osteogenic medium had more significantly elevated activity than spheroids growth in the regular culture medium. Expression of ALP marker at the mRNA level at 14 days of culture determined by qPCR showed that ALP reaches its highest expression in the presence of osteogenic factors as 0.4-fold more elevated than the control media (Supplementary Figure S1). These differences in the assay showed that the effect of the osteogenic factors has a positive impact on the early phase of osteoblast differentiation, and this is in agreement with studies that report that ALP is a marker of osteoblasts phenotype, that confirms the maturation period and the beginning of osteoblasts differentiation and extracellular matrix mineralization (Wennberg et al., 2000; Kim et al., 2012; Martins et al., 2014; Sharma et al., 2014).

Collagen is the most abundant bone matrix protein, constituting up to 90%, and play an essential role in the initial regulation of bone tissue formation and is considered as an early marker of osteoblast differentiation, which in turn is shown to be necessary for subsequent bone markers expressions and biomineralization (Olsen et al., 2000). Our results of the analysis of the expression of Col 1 at mRNA level showed a significant increase when 3D hFOB spheroid was cultured onto osteogenic media at 14 days around 0.7-fold against control media (Supplementary Figure S1). This difference in the Col 1 marker expression could be because the osteogenic media contain ascorbic acid (AA). Several studies have reported that ascorbic acid acts as a cofactor for essential enzymes in the biosynthesis of collagen, and the extracellular matrix accumulation of Col I is strongly dependent on AA because it plays a predominant role in the stabilization of cell-synthesized collagen matrix (Qiao et al., 2009; Langenbach and Handschel, 2013; Unal et al., 2018; Maione-Silva et al., 2019).

In the biomineralization process, osteoblasts secret Col 1 acts as a template for calcium and supports the deposition of the extracellular matrix mineralization. The mineralization of the 3D hFOB spheroids culture on magnetic levitation was revealed by alizarin red staining (ARS). As observed in light micrographs of the 3D hFOB spheroids without osteogenic factors, the extracellular matrix showed a slight red positive signal in the cell aggregates or clusters and at the periphery of the spheroids (Figure 2C,D). In contrast, 3D hFOB spheroids culture in the presence of osteogenic factors showed an enhanced alizarin red staining, leading to the formation of denser and positively stained clusters at the periphery of the spheroids (Figure 2E,F). Besides, ARS staining binds selectively to calcium salts, infers the mineralization, and allows the quantification because the dye can be extracted and read from the stained layers (Gregory et al., 2004). As shown in Figure 2G, the calcium content assay revealed a significant increase in the mineralization phase after 14 days onto the 3D hFOB spheroid under the presence of osteogenic factors in comparison with the 3D hFOB spheroid without it. These high levels could indicate that the osteoblast-like cells have a positive cellular environment where cell-cell and cell-ECM interactions are appropriate to induce the osteogenesis in the 3D structure and regulate the osteoblasts function, playing an essential role in determining mineralization capacity (Wang et al., 2009; Boonrungsiman et al., 2012). Moreover, it is reported that cell condensation plays a significant role in osteogenesis differentiation and 3D spheroid models enabled to evoke the cell aggregation and condensation (Kim and Adachi, 2019).

**FIGURE 3:**
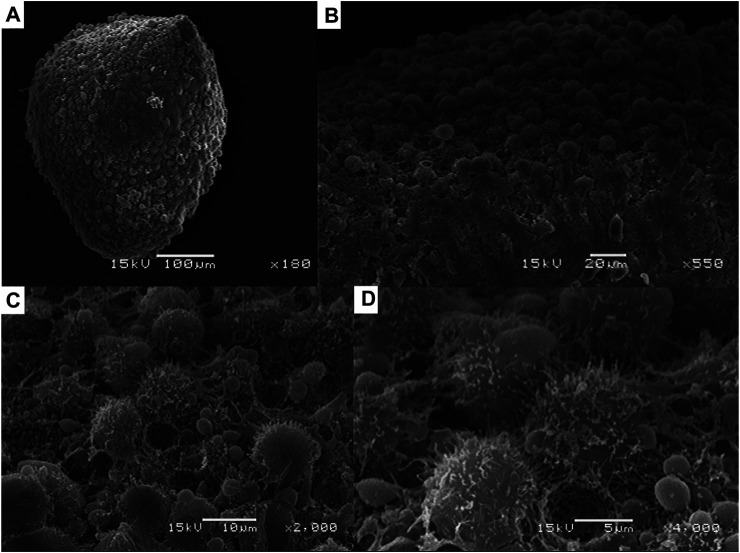
**(A)** Examination of the morphology and structural integrity of the 3D hFOB spheroid by scanning electron microscopy without the presence of osteogenic factors after 14 days of cultured under magnetic levitation. **(B)** Close-up of the 3D hFOB spheroid where could reveal the surface characteristics of the aggregates osteoblasts that form the spheroid. **(C, D)** High magnification images of cell aggregates where could be seen cell to cell boundaries.

**FIGURE 4:**
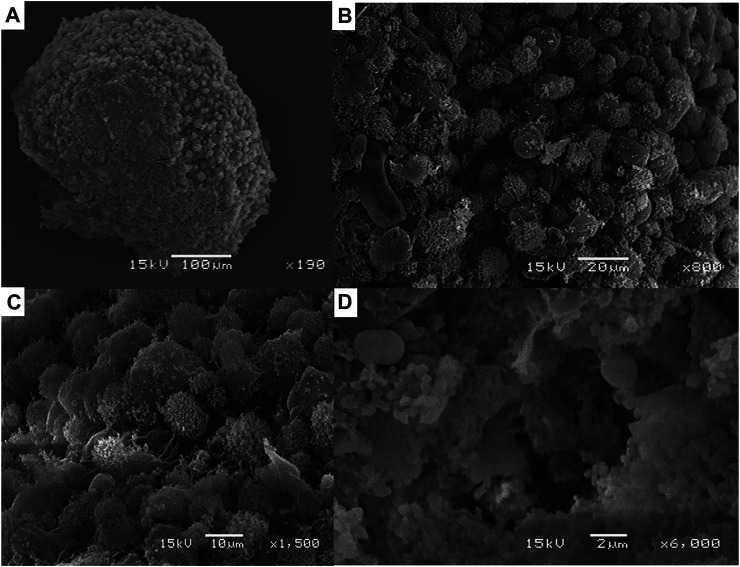
**(A)** Morphology of the 3D hFOB spheroid obtained by scanning electron microscopy with the presence of osteogenic factors in the cell culture media incubated for14 days under magnetic levitation system. **(B)** High magnification of the 3D hFOB spheroid showed that osteoblasts cells are highly cohesive showing that magnetic force-mediated cell clustering. **(C)** Close-up of the compacted cells where could be seen defined cell-cell interaction of the compacted osteoblast cells. **(D)** Presence of granular material suggestive of mineral extracellular material deposition by the hFOB cells.

The analysis of the 3D spheroid morphology, formed by hFOB cells under magnetic levitation and observed by SEM, showed a regular and stable aggregation of the osteoblast cells that allow forming a compact surface spheroid with closed cell-cell contact interactions between them (Figures 3, 4). These results are in agreement with the behavior that osteoblast cell-cell attachment is vital for survival, and in our study, we reported the expression of Col I as a specific extracellular matrix protein that could allow the cell adhesion under the magnetic levitation system as an essential process for influenced osteoblast cell morphology, proliferation, and differentiation. Moreover, cell-matrix adhesions control cell behavior on 3D, which is generally mediated through a specific class of transmembrane adhesion receptors known as integrin. Using the analysis of the expression of integrin β-1, we observed that the mRNA level was expressed in both 3D hFOB spheroid cultured with and without the presence of osteogenic media (Supplementary Figure S1). The association of osteoblast with the extracellular matrix through integrins may be restricted to specific stages of osteoblasts or mineralized matrix maturation where it is reported that the β1 integrins appear to have the major functional role with a high affinity for the Gly-Glu-Arg (GER) motif in collagen type 1 (Bennett et al., 2001; Gronthos et al., 1997). Thus, from the results is possible to correlate the biological processes in the formation of the 3D hFOB spheroid under a magnetic levitation system; first attachment between cells could be mediated by non-receptor via electrostatic bonding; however, this cell adhesion does not give any signal to cells only allow to aggregate, moreover the continue cell-cell attachment prompt the microenvironment to play a vital role to regulate the secretion of collagen type 1 as ECM proteins by the osteoblasts and function as ligand cue to cells that allow the integrin β1 binding that leads to formed dot-like focal adhesion that communicated with the activation of intracellular signal transduction pathways with structural and signaling molecules, such as tallin, α-actinin, filamin, paxillin or vinculin, which, in turn, contribute to the regulation of cell-cell cytoskeletal organization, migration, proliferation, and differentiation (Anselme, 2000; Moreno-Layseca and Streuli, 2014; Cruz-Maya et al., 2018). This stable adherence is confirmed by a higher magnification image where the strong interactions between osteoblasts cells, located at the external layer, and demonstrated a highly dense cellular network (Figures 3B, 4B). Furthermore, the majority of osteoblast cell morphology showed numerous cytoplasmic and cell membrane protrusions that suggest close contacted between neighbor cells; and the displayed of a complex three-dimensional network of microvilli or small filamentous structures indicative of extracellular matrix deposition was also presented (Figures 3C,D, 4C). Nevertheless, the extracellular matrix of 3D hFOB spheroid cultivated in osteogenic factors exhibited the presence of an intercellular space rich in a granular material, suggesting an increased mineral deposition, and confirmed by the ARS staining assay (Figure 4D). In this regard, the morphology of tissue spheroids could represent a compact 3D tissue construct, and this is in agreement with previous reports on 3D spheroid aggregation, histology, and computer simulation analysis of multicellular spheroids (Alno et al., 2010; de Barros et al., 2010; Edmondson et al., 2014; Parfenov et al., 2018). Nonetheless, our study indicates that the magnetic levitation approach is a low-cost system that allows rapid 3D human fetal osteoblast spheroid formation, driven mainly by the presence of the magnetic field, favoring the easy handle of the 3D spheroid and, most important without damage to the osteoblasts cell population.

## Conclusion

In this work, our data indicate that magnetic levitation systems using human fetal osteoblast cell lines for 3D multicellular spheroid formation can be easily reproduced; and emphasizes the great potential to perform a microtissue-like model. In this context, the present results indicate that 3D hFOB spheroids developed by magnetic levitation systems can be evaluated for biocompatibility and show that long-term culture is feasible. Overall, the *in vitro* characterization showed that 3D hFOB spheroid cultured by magnetic levitation does not reduce cell viability, produce a compact spheroid morphology and provide evidence of osteogenic differentiation. Moreover, 3D hFOB spheroid showed a fully formed cell-cell and cell-extracellular matrix interactions playing a role in the required physiological response by osteoblasts of the cells attachment for continuing to the osteoblast maturation and production of the deposition of the mineralized extracellular matrix. However, further functional studies are needed to characterize the regulation of the extracellular environment concerning the osteogenesis differentiation and elucidated in detail *in vitro* or *in vivo* the molecular formation of bone achieved using the 3D spheroids culture system. Hence, all *in vitro* results suggested that magnetic levitation culture constitute a very innovative and promising technology that allows the generation of 3D stable osteoblast spheroid that could be a profitable strategy for tissue engineering application, biofabrication to re-establish native tissue architecture and open the potential in bone regenerative medicine to treat musculoskeletal defects based on spheroids.

## Data Availability

The original contributions presented in the study are included in the article/Supplementary Material, further inquiries can be directed to the corresponding author.
