# Supplementary material for: Case Report: Formation of 3D Osteoblast Spheroid Under Magnetic Levitation for Bone Tissue Engineering
Source: Front Mol Biosci. 2021 Jun 21;8:672518. doi: 10.3389/fmolb.2021.672518 (PMC8255365; doi:10.3389/fmolb.2021.672518)
Supplement: Supplementary file 1 [file DataSheet1.docx]

**Supplementary Figure**


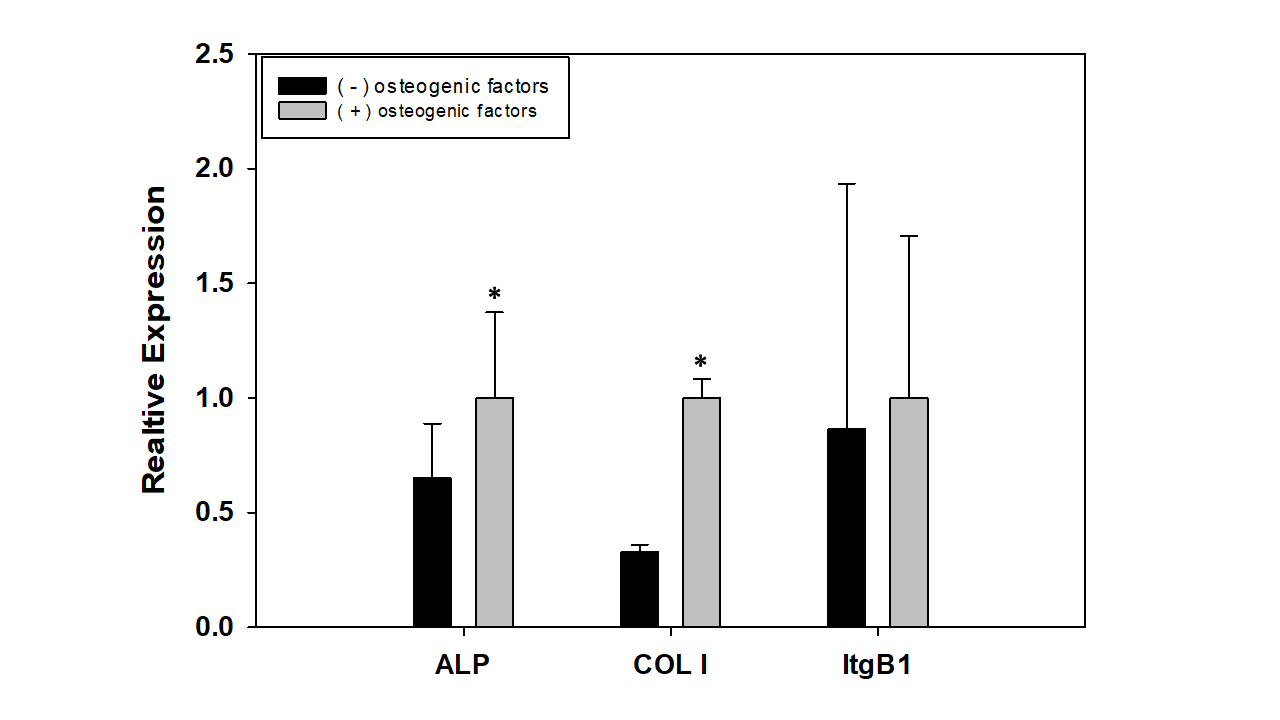


Figure S1. Real-time quantitative PCR evaluation of the expression of representative genes of hFOB 3D spheroids at 14 days of culture under magnetic levitation system with and without the presence of osteogenic media. The histogram showed the high level of alkaline phosphatase (ALP) and collagen type 1 (COL I) transcripts when compared to control. Integrin β1 (Itgβ1) showed similar expression in both 3D hFOB spheroids. Results of the expression of representative target genes were normalized against GAPDH. Data represent the mean values of performed triplicates per test sample. Asterisks ( * ) indicate significant differences at p-value < 0.05 by Student’s t test.
